# A motile doublet form of *Salmonella* Typhimurium diversifies target search behavior at the epithelial surface

**DOI:** 10.1111/mmi.14898

**Published:** 2022-04-12

**Authors:** Viktor Ek, Stefan A. Fattinger, Alexandra Florbrant, Wolf‐Dietrich Hardt, Maria Letizia Di Martino, Jens Eriksson, Mikael E. Sellin

**Affiliations:** ^1^ Science for Life Laboratory, Department of Medical Biochemistry and Microbiology Uppsala University Uppsala Sweden; ^2^ Institute of Microbiology, Department of Biology ETH Zurich Zurich Switzerland

**Keywords:** bacterial infection, bacterial morphology, epithelium, flagella, swim pattern

## Abstract

The behaviors of infectious bacteria are commonly studied in bulk. This is effective to define the general properties of a given isolate, but insufficient to resolve subpopulations and unique single‐microbe behaviors within the bacterial pool. We here employ microscopy to study single‐bacterium characteristics among *Salmonella enterica* serovar Typhimurium (*S*.Tm), as they prepare for and launch invasion of epithelial host cells. We find that during the bacterial growth cycle, *S*.Tm populations switch gradually from fast planktonic growth to a host cell‐invasive phenotype, characterized by flagellar motility and expression of the Type‐three‐secretion‐system‐1. The indistinct nature of this shift leads to the establishment of a transient subpopulation of *S*.Tm “doublets”—waist‐bearing bacteria anticipating cell division—which simultaneously express host cell invasion machinery. In epithelial cell culture infections, these *S*.Tm doublets outperform their “singlet” brethren and represent a hyperinvasive subpopulation. Atop both glass and enteroid‐derived monolayers, doublets swim along markedly straighter trajectories than singlets, thereby diversifying search patterns and improving the surface exploration capacity of the total bacterial population. The straighter swimming, combined with an enhanced cell‐adhesion propensity, suffices to account for the hyperinvasive doublet phenotype. This work highlights bacterial cell length heterogeneity as a key determinant of target search patterns atop epithelia.

## Graphical abstract

Part of a *Salmonella* Typhimurium (*S*.Tm) population is able to remain motile and invasive while mid‐cell division. These “doublets” swim markedly straighter than the rest of the *S*.Tm population, which diversifies target search patterns atop epithelia, thus improving the surface exploration capacity of the bacterial population as a whole. This adds bacterial cell length heterogeneity as a virulence determinant and emphasizes the need for a close‐up view to fully understand bacterial pathogenesis.

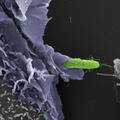

## 1 INTRODUCTION

Following oral infection, enteropathogenic bacteria such as *Salmonella enterica* serovar Typhimurium (*S*.Tm) can grow and divide planktonically in the intestinal lumen, and also colonize the mucosal epithelium. Luminal expansion depends on access to nutrients, successful competition with the resident microbiota, and the activity of immune defenses within the infected host (McLaughlin et al., 2019; Nguyen et al., 2020; Stecher et al., 2007; Winter et al., 2010). Part of the population invades intestinal epithelial cells, giving *S*.Tm access to an additional, intracellular replication niche (Castanheira & García‐del Portillo, 2017; Fattinger et al., 2021). Moreover, cycles of epithelial cell invasion, intracellular expansion, and reseeding of the gut lumen by epithelium‐lodged *S*.Tm may further bolster the gut colonization effort (Chong et al., 2021; Geiser et al., 2021; Knodler et al., 2010). The invasion process also elicits an acute inflammatory response of the mucosal tissue that thwarts the homeostatic balance of the gut ecosystem, in favor of *S*.Tm. Despite that the inflammatory response eradicates a large fraction of the total pathogen population (Maier et al., 2014), surviving *S*.Tm gains a competitive edge against the microbiota within the inflamed gut, thereby achieving long‐term colonization (Stecher et al., 2007; Winter et al., 2010). Hence, extracellular growth and bacterial invasion of epithelial cells constitute two key events of the *S*.Tm infection cycle, which are intimately linked in several ways.

Enteropathogens express specific virulence factors to colonize the gut epithelium. In the case of *S*.Tm, ~2–8 evenly distributed (peritrichous) flagella allow the bacteria to explore breaches in the intestinal mucus, reach the epithelial surface, and probe it for suitable invasion sites, through near‐surface swimming (Furter et al., 2019; Misselwitz et al., 2012). Areas of surface unevenness, e.g., at cell–cell junctions, make up particular hot spots for bacterial adhesion and subsequent epithelial cell invasion (Fattinger et al., 2020; Friedlander et al., 2013; Misselwitz et al., 2012). Additionally, the flagella (Crawford et al., 2010; Horstmann et al., 2020; Wolfson et al., 2020), as well as dedicated adhesins (Gerlach et al., 2007; Li et al., 2019; Suwandi et al., 2019), aid in the initial binding of *S*.Tm to the target cell (reviewed in Rehman et al., 2019; Wagner & Hensel, 2011). A Type‐III‐Secretion‐System (TTSS‐1), encoded by *Salmonella* pathogenicity island‐1 (SPI‐1), secures this interaction by docking its translocon complex directly into the host cell membrane (Collazo & Galán, 1997; Kubori et al., 1998; Lara‐Tejero & Galán, 2009; Misselwitz et al., 2011). This sparks the transfer of a set of TTSS‐1 effectors into the target cell to activate host cell actin‐regulatory proteins, including e.g., Rho‐ and Arf‐family GTPases, and formins (Davidson et al., 2015; Hardt et al., 1998; Patel & Galán, 2006; Stender et al., 2000; Truong et al., 2013). In cultured epithelial cell lines, the effectors elicit expansive and dynamic membrane ruffles for *S*.Tm uptake, whereas the corresponding entry structures appear less pronounced in the strictly polarized epithelium of the intact gut (Fattinger et al., 2020). Nevertheless, efficient *Salmonella* invasion of epithelial cells has been demonstrated to occur in a TTSS‐1‐dependent manner across a variety of host species, bacterial serovars, and in a multitude of different tissue culture models (Barthel et al., 2003; Di Martino et al., 2019; Fattinger et al., 2020; Geiser et al., 2021; Lhocine et al., 2015; Zhang et al., 2018).

Expression of the *S*.Tm virulence factors that drive epithelial cell invasion is costly and tightly regulated in response to environmental cues (Knodler et al., 2002; Kröger et al., 2013; Sturm et al., 2011). When nutrients are in excess, *S*.Tm sustains fast, exponential growth to expand the population size, but typically exhibits limited expression of motility‐ and SPI‐1‐associated genes (Kröger et al., 2013). By contrast, SPI‐1 gene expression and mounting of the TTSS‐1 apparatus occur when nutrients are scarce, observed at the late exponential–early stationary phase transition of an *S*.Tm broth culture (Kröger et al., 2013). Moreover, several other cues linked to stress, e.g., hyperosmotic‐ or oxygen‐shock, as well as growth in anaerobic environments, have been shown to fuel expression of the virulence machinery (Bajaj et al., 1996; Ibarra et al., 2010; Kröger et al., 2013). A simplistic interpretation of these findings is that *S*.Tm switches between two states in the infected gut, i.e., a rapidly growing but non‐invasive state under favorable conditions, and a non‐growing but epithelial cell‐invasive state under unfavorable conditions. Here, we explore the relationship and transition between these states.

Traditionally, studies of how *S*.Tm and related pathogens colonize epithelial cells have employed bulk infection experiments, e.g., the widely used gentamycin protection assay (Di Martino et al., 2019; Steele‐Mortimer, 2007). These assays make it possible to quantify and compare the invasion efficiencies of different strains, scored at the level of population averages. However, bulk assays fail to resolve the presence of subpopulations and unique single‐microbe behaviors within a given bacterial pool. More recently, high‐resolution microscopy and flow cytometry approaches have shown that divergent single‐cell behaviors are indeed commonplace among bacterial pathogens. For example, also under virulence‐inducing conditions, subpopulations of *S*.Tm that either express or do not express SPI‐1 genes coexist within the same pathogen population—a phenomenon referred to as bistable expression (Sánchez‐Romero & Casadesús, 2018; Sturm et al., 2011). This extends also to, e.g., the flagella, which cooperate with SPI‐1, hinting at a complex mosaic of expression patterns regulating virulence (Sánchez‐Romero & Casadesús, 2021). Although single‐cell approaches are increasingly utilized (reviewed by Davis & Isberg, 2016), our understanding of heterogeneous bacterium‐to‐bacterium variation within a pathogen population, and its potential impact on host cell‐invasive behavior, still remains limited.

In this work, a series of microscopy experiments were used to probe single‐bacterium characteristics among *S*.Tm as they seek out and invade epithelial cells. We find that a significant overlap exists between the bacterial exit from fast growth and the induction of the *S*.Tm host cell invasion machinery (i.e., flagella and TTSS‐1). This results in the formation of a transient, but substantial, subpopulation of *S*.Tm “doublets”—bacteria anticipating cell division—that simultaneously exhibit both motility and SPI‐1 expression. These virulent *S*.Tm doublets swim along straighter trajectories than the corresponding “singlets,” thereby diversifying swim behavior and the exploratory ability of the bacterial population during near‐surface swimming. The straight swimming, combined with an enhanced host cell surface‐binding ability, also makes doublets superior first invaders that promote early *S*.Tm epithelial cell colonization.

## 2 RESULTS

### 2.1 High frequency of morphological “doublets” among epithelial cell‐invading *S*.Tm

**FIGURE 1:**
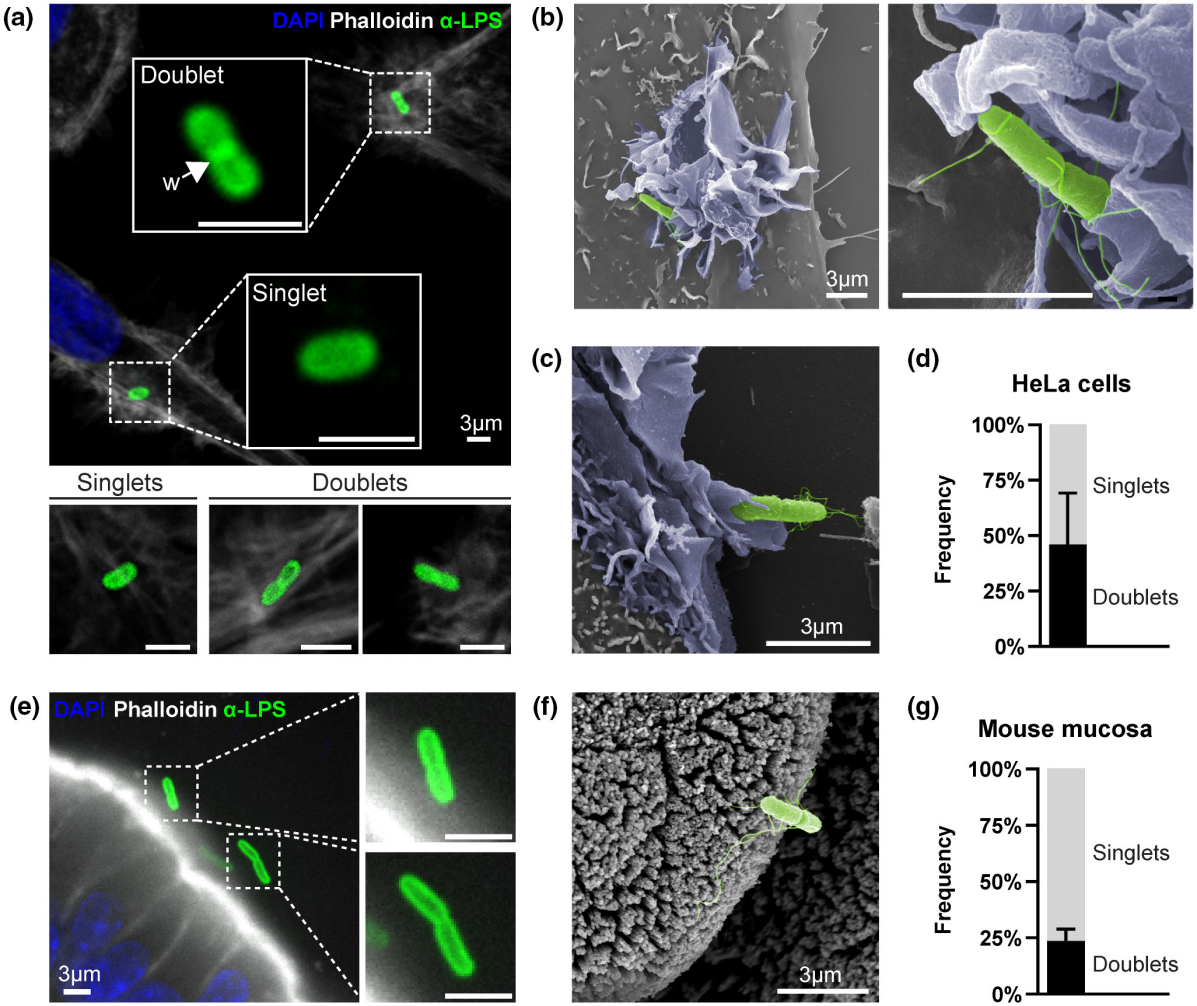
Abundance of *S*.Tm doublets among bacteria adhering to and invading epithelial cells. (a) Representative fluorescence micrographs of HeLa cells infected with *S*.Tm^*wt*^ (MOI = 10), resulting in characteristic actin ruffles around the invading bacteria. Enlarged are examples of bacteria identified as “singlets” (~2 μm, archetype) or “doublets” (~3–4 μm long, criterion: with visible waist). “W” and arrow denote the waist of a doublet. Cultures were infected for 7 min before fixation and staining with DAPI (blue), Alexa Fluor 488 Phalloidin (gray), and anti‐LPS antibodies (green). (b‐c) Representative SEM micrographs of *S*.Tm^*wt*^‐infected HeLa (b) and mIC‐c_12_ (c) cells, illustrating doublets within entry ruffles (MOI = 400). Fixation was done at 10 min p.i. Ruffles (blue) and bacteria (green) were manually pseudo‐colored during post‐processing. (d) Quantification of the frequency of singlets and doublets within entry ruffles in HeLa cells infected with *S*.Tm^*wt*^ as in A. Only ruffles containing single bacteria were quantified. Data are shown as mean ± SD, representative for three independent experiments (total ruffles analyzed *n =* 259). (e) Representative fluorescence micrograph of cecal tissue from an *Nlrc4* ^*−/−*^ mouse infected with 5 × 10^7^ colony forming units (CFUs) of *S*.Tm^*wt*^ for 9 h, followed by extensive washing of tissue and staining post‐fixation with DAPI (blue), Alexa Fluor 488 Phalloidin (gray), and anti‐LPS antibodies (green). Enlarged are examples of *S*.Tm doublets at the epithelial surface. (f) Representative SEM micrograph of infected WT mouse cecal tissue as in e, harvested at 8 h p.i. The bacterium has been manually pseudo‐colored during post‐processing (green). (g) Quantification of the frequency of singlets and doublets remaining adhered to the mouse cecal mucosa after extensive washing (as in e). Data are shown as mean ± SD, representative for three mice (total bacteria analyzed *n* = 520). Scale bars in all panels: 3 μm

When added to epithelial cells in culture, *S*.Tm within minutes reach the host cell surface by flagellar motility, adhere to the cell membrane by the combined action of adhesins and TTSS‐1, and elicit ruffle‐mediated entry (reviewed by Fattinger et al., 2021). We imaged *S*.Tm invasion events in human (HeLa) and mouse (m‐IC_cl2_) epithelial cell lines by high‐resolution confocal fluorescence and scanning electron microscopy (SEM). In addition to the bacilli‐shaped ~2‐μm long bacteria (“singlets”), a prominent category of longer bacteria (typically 3–4 μm) with a visible waist was frequently observed among the *S*.Tm captured in the process of epithelial cell invasion (Figure 1a–c). These features are consistent with bacteria actively growing in preparation for daughter cell separation (Cooper, 1988). Using the waist criterion (see Figure S1a,b and experimental procedures for further details), we could classify and quantify this category of *S*.Tm, hereafter denoted “doublet(s)” (Figure 1a–c). By categorizing invading *S*.Tm as either singlets or doublets, we found that a striking 46.0 ± 23.1% of all *S*.Tm observed to trigger entry ruffles in HeLa cells were doublets (Figure 1d).

To assess if doublets could also be found among epithelium‐interacting *S*.Tm in the infected gut in vivo, we per‐orally infected C57BL/6 mice according to a well‐established protocol (Barthel et al., 2003). At 8–9 h post‐infection (p.i.) the cecae were excised and the mucosal tissue was washed extensively to remove luminal bacteria. Again, an inspection of epithelium‐associated *S*.Tm populations revealed both singlets and doublets (Figure 1e,f). The size of the doublet category varied somewhat between animals but was estimated to be 23.5 ± 5.4% (Figure 1g). To further generalize the findings, we scrutinized several previous studies of *S*.Tm epithelial cell invasion across different settings and by different research groups. Many reports contained images with clearly distinguishable *S*.Tm doublets interacting with or invading epithelial cells (Brooks et al., 2017; Fattinger et al., 2020; Fredlund et al., 2018; Gerlach et al., 2007; Humphreys et al., 2012; Suwandi et al., 2019). Therefore, it appears an unappreciated doublet form of *S*.Tm is prevalent during epithelial cell binding and invasion in a variety of experimental settings.

### 2.2 *Salmonella* Typhimurium doublets are transient, but hyperinvasive under flagella‐ and TTSS‐1‐inducing conditions

In broth culture, bacterial growth rate peaks in exponential phase, whereas the expression of virulence factors required for *S*.Tm to adhere to and invade host cells (particularly TTSS‐1) coincides with slowed growth and the transition into stationary phase (Kröger et al., 2013). Motivated by the unexpectedly high frequency of doublets in the host cell‐invading population (Figure 1), we next explored the interdependencies between the growth phase, the frequency of doublets, and the expression of virulence machinery required for *S*.Tm epithelial cell invasion.

**FIGURE 2:**
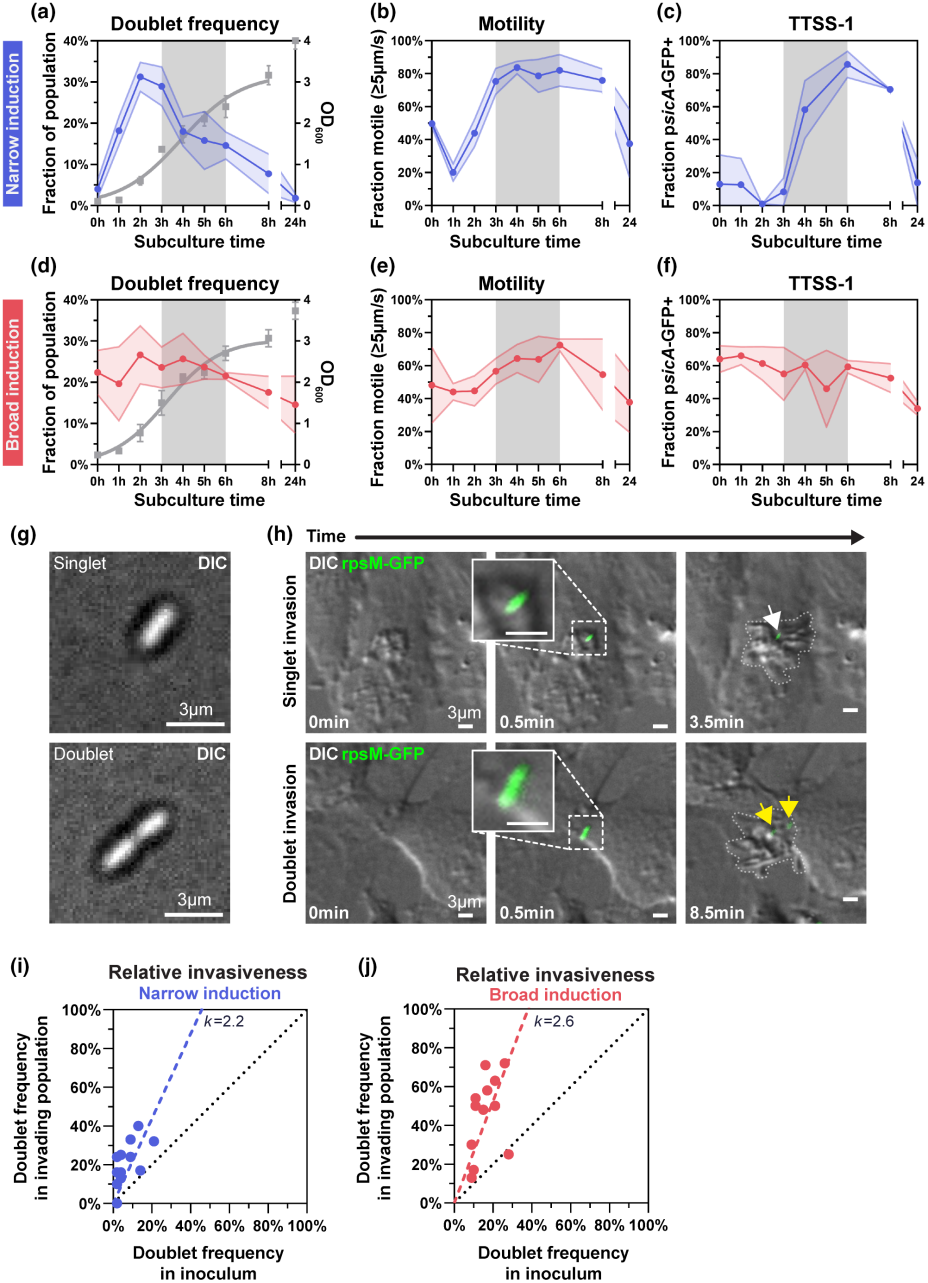
*S*.Tm doublets promote epithelial cell invasion under virulence‐inducing conditions. (a–f) Variation in doublet frequency, motility, and expression of TTSS‐1 across different time‐points post subculture (p.sc.), under narrow (a–c) or broad (d–f) induction conditions, quantified by single‐cell microscopy. Gray shading denotes the late exponential phase–early stationary phase transition (time‐points used in subsequent experiments). Each panel shows data as mean ± SD of three experiments (very low SD not visible for some points). (a) Quantification of doublets in *S*.Tm^*wt*^ cultures grown ON, sub‐cultured 1:100 in LB medium (narrow induction condition), and incubated 0–24 h before imaging. Data expressed as a fraction of total population (blue curve, left y‐axis). The growth curve is represented by a sigmoidal fit of OD_600_ measurements (gray curve, right y‐axis; fit excludes the 24 h time‐point). (b) Fraction of motile bacteria (>5 μm/s) observed under conditions as in a, generated by single‐particle tracking. (c) The SPI‐1 reporter strain *S*.Tm/p*sicA*‐GFP was grown as in a and the frequency of GFP‐expressing bacteria was quantified by microscopy. (d–f) Similar data as in a‐c but acquired for *S*.Tm cultures grown under the broad induction condition (12 h ON followed by 1:20 subculture in LB/0.3 M NaCl). (g) Example DIC images of a singlet and a doublet in the inoculum used for infections. (h) Representative time series of HeLa cells infected with an *S*.Tm/p*rpsM*‐GFPmut2 constitutive reporter strain. Cells in greyscale, bacteria (GFP) in green. Arrows denote examples of invading singlet (white arrow) and doublet *S*.Tm (yellow arrow), the latter subsequently dividing into two daughter cells. Entry ruffles are delimited by dotted lines (right‐most panel). The time indicated in minutes. (i and j) Quantification of the frequency of *S*.Tm doublets in the inoculum (x‐axis) vs. in the corresponding ruffle‐inducing population in HeLa cells during the first 30 min of co‐incubation (y‐axis). The black dotted line illustrates a theoretical 1:1 ratio (*k* = 1). For i and j, each graph shows data for 3–6 h subcultures used as inocula, pooled from three independent experiments (total *n* = 12 infections). Data from different subcultures are shown as filled circles and linear regression as dashed lines for the narrow (i; *k* = 2.2) and broad (j; *k =* 2.6) induction condition, respectively. Scale bars in all panels: 3 μm

The degree and timing of virulence gene expression will differ depending on the protocol used to culture the bacterial inoculum. Therefore, we conducted our analyses under two commonly used growth conditions, resulting in either a growth phase‐selective induction or a more constant and broader induction, of virulence traits (henceforth referred to as “narrow” and “broad” induction condition, respectively). When a 24 h overnight (ON) culture of *S*.Tm was sub‐cultured 1:100 in LB (“narrow” induction condition), the culture entered exponential phase at ~1 h post‐subculture (p.sc.), maintained fast growth between 1 and 3 h p.sc., and thereafter decelerated growth, as measured by optical density (OD) (Figure 2a, right y‐axis). In line with that fast‐growing *S*.Tm cultures should contain a high percentage of bacteria anticipating daughter cell separation, the frequency of doublets peaked in exponential phase, with up to ~32% of all bacterial bodies mapping to this category (Figure 2a; bacterial length distributions provided in Figure S1c). Importantly, after exit from fast exponential growth (≥3 h p.sc.), doublets remained prevalent (~10–20% of total *S*.Tm bodies at 4‐6 h p.sc.; Figure 2a). To survey how the doublet frequency related to flagellar motility and TTSS‐1 expression (i.e., the two main requirements for epithelial cell invasion competence), we scored the frequency of motile and TTSS‐1+ *S*.Tm across the broth culture cycle in parallel (Figure 2b,c). Motility was assessed by differential interference contrast (DIC) time‐lapse microscopy followed by single‐particle tracking (Video S1; Figure S2), whereas for SPI‐1/TTSS‐1 expression we employed a p*sicA*‐GFP reporter (Figure S3a,b; Sturm et al., 2011). This analysis confirmed that the majority of *S*.Tm turned motile (defined as having a speed of >5 μm/second) after their exit from fast exponential growth (Figure 2b; ≥3 h p.sc.). Similarly, TTSS‐1 expression remained low throughout the first 3 h p.sc., but increased to ~80% TTSS‐1+ bacteria between 3‐6 h p.sc. (Figure 2c). We conclude that under the narrow induction condition, *S*.Tm cultures at ~3‐6 h p.sc. feature both a considerable doublet fraction and express the virulence machinery required for epithelial cell invasion.

The second growth condition applied a common protocol to robustly induce the *S*.Tm invasion machinery via a shorter 12 h ON incubation step in hypertonic LB (LB/0.3 M NaCl), followed by sub‐culturing at a 1:20 dilution in the same LB/0.3 M NaCl for a few h (“broad” induction condition; Fattinger et al., 2020; García‐Calderón et al., 2007; Song et al., 2004). We repeated the analysis for this condition (Figure 2d–f). As anticipated from the short ON step of the starter culture, the modest subculture dilution, and the effects of the hypertonic broth, the differences in doublet frequency, percentage motile, and percentage TTSS‐1+ *S*.Tm were less distinct between the phases, when compared to the narrow induction condition (Figure 2d–f; compare to Figure 2a–c). Nevertheless, we again observed the existence of a substantial doublet subpopulation (~20–25% of all *S*.Tm bodies) at phases coinciding with a high frequency of motile and TTSS‐1+ *S*.Tm—e.g., in the 3–6 h p.sc. window used in prior studies of host cell invasion (Figure 2d–f; Fattinger et al., 2020; Lee et al., 1992; Song et al., 2004; Steele‐Mortimer et al., 1999).

The high frequency of doublets observed in invasion ruffles (Figure 1d) led us to hypothesize that doublets may represent a hyperinvasive subpopulation of *S*.Tm present in inocula. To test this hypothesis, we employed time‐lapse DIC and fluorescence microscopy to quantify singlets and doublets in the bacterial inoculum used to infect HeLa cells, as well as among the “first invaders”, i.e., those *S*.Tm eliciting entry ruffles promptly after the addition of the inoculum to the cell culture (Figure 2g,h; Video S2). Constitutively fluorescent *S*.Tm/p*rpsM*‐GFP were cultured under either the narrow or broad induction condition, and multiple real‐time infection experiments were executed using the 3–6 h sub‐cultures (i.e., bacteria harvested at 3, 4, 5, or 6 h p.sc.; gray shading in Figure 2a–f) as inocula. Strikingly, for both induction protocols, doublets were markedly overrepresented in the epithelial cell‐invading *S*.Tm population, as compared to their frequency in the corresponding inoculum (Figure 2i,j). This could not be explained by an up‐growth of doublets in the tissue culture medium, since doublet frequency stayed the same or even dropped in the planktonic *S*.Tm population over the 30‐min imaging period (Figure S3c–e). Linear regression including all replicates suggested doublets to be ~2.2‐fold (narrow induction condition; Figure 2i) to ~2.6‐fold (broad induction condition; Figure 2j) more likely than singlets to invade epithelial cells in culture.

Taken together, these data suggest that motile and TTSS‐1+ doublets represent a transient, but epithelial cell‐hyperinvasive, subpopulation of *S*.Tm.

### 2.3 Singlets and doublets exhibit similar TTSS‐1 activity, while doublets adhere modestly better to host cells

**FIGURE 3:**
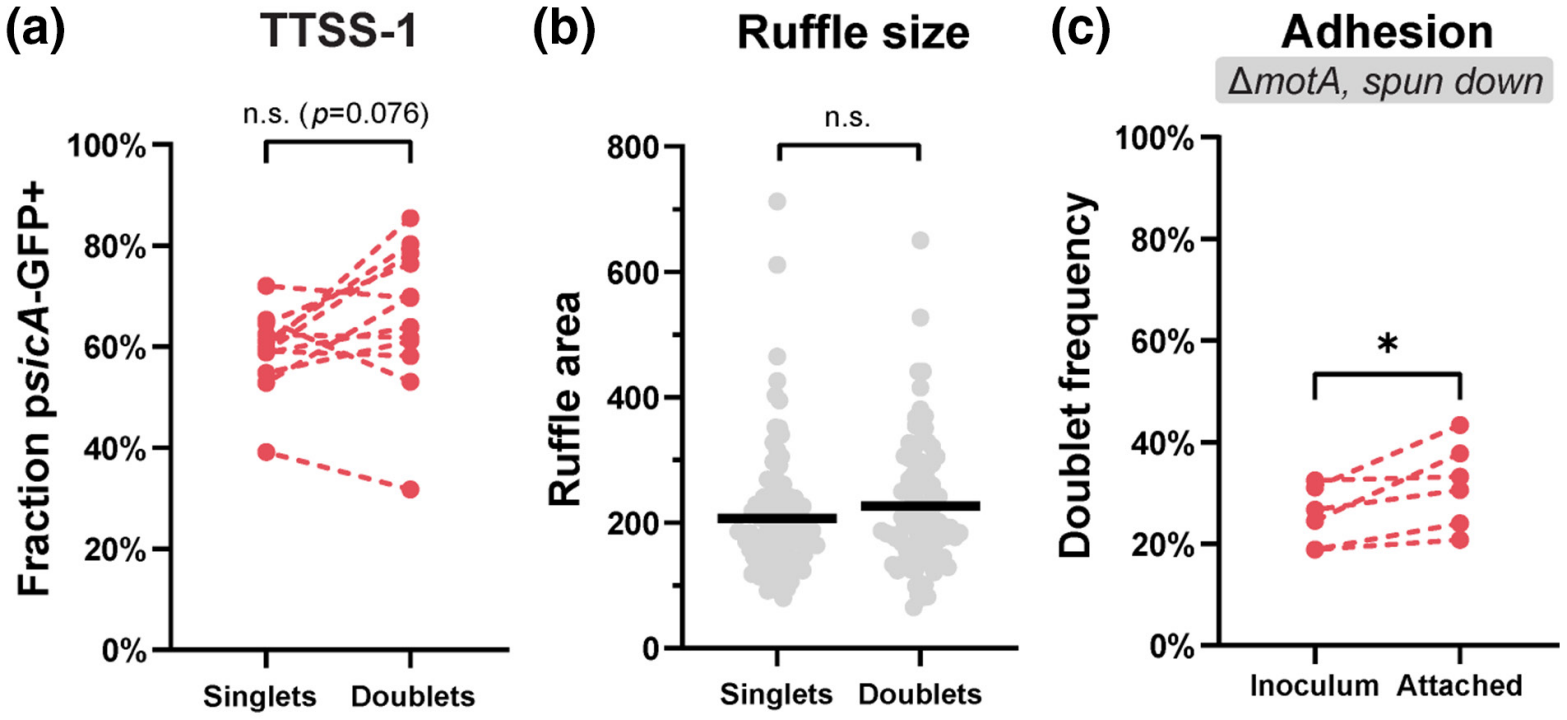
Differences in TTSS‐1 expression and adhesion capacity are insufficient to explain *S*.Tm doublet hyperinvasiveness. (a) Comparison between *S*.Tm singlet and doublet subpopulations in the expression of TTSS‐1, using the p*sicA*‐GFP reporter strain cultured under the broad induction condition and analyzed at 3‐6 h p.sc. (sourced from the same experiments as Figure 2f). Data are shown as paired comparisons for *n* = 12 replicates, pooled from three independent experiments. (b) Quantification of entry ruffle area upon invasion of HeLa cells by singlets and doublets. Filled dots show individual ruffles, lines represent means. Data pooled from three infection experiments, using *S*.Tm^*wt*^ grown under the broad induction condition for 4 h as inoculum (total ruffles analyzed *n* = 200). (c) Comparison of doublet frequency in inocula and the population attaching to HeLa cells. HeLa cells were treated with Cytochalasin D and co‐incubated for 10 min with pFPV‐mCherry‐carrying inocula (broad induction condition, 4 h p.sc.) of a non‐motile strain (*S*.Tm^*ΔmotA*^). Cells were centrifuged to promote contact, washed, and the attached bacteria categorized. Data are shown as paired comparisons for *n* = 6 independent experiments. Statistical analyses via paired *t* test (a and c) or Mann–Whitney *U* test (b) (*n.s*.: non‐significant; *: *p <* 0.05)

Wild‐type *S*.Tm invasion of epithelial cells in culture occurs through TTSS‐1‐induced entry ruffles (Figure 1). It remained conceivable that doublets would be overrepresented among invading bacteria because of more frequent TTSS‐1 expression. However, pair‐wise comparison of TTSS‐1+ singlets and doublets within the relevant growth phase (3‐6 h p.sc.) resolved no difference in the frequency of TTSS‐1‐equipped bacteria under either of the two growth conditions (Figure 3a; Figure S4a). Furthermore, singlet and doublet categories elicited HeLa cell ruffles with a similar mean area of ~200μm^2^, with only a weak trend toward doublets being more likely to elicit larger ruffles (Figure 3b).

Entry is preceded by a binding step that involves reversible interactions via dedicated adhesins and flagella (Gerlach et al., 2007; Horstmann et al., 2020; Misselwitz et al., 2011), and stable docking via TTSS‐1 (Lara‐Tejero & Galán, 2009; Misselwitz et al., 2011). We next explored the binding capacity of singlets and doublets by incubating HeLa cells pre‐treated with Cytochalasin D (blocks actin‐dependent uptake). To abolish any impact of flagellar motility, these experiments employed a MotA‐deleted strain (*S*.Tm^*ΔmotA*^; retains structural flagella, but lacks motor complex function; Yamaguchi et al., 1986). Quantification of the doublet frequency in the host cell‐binding population revealed only a nominal enrichment of doublets (~1.2‐fold, Figure 3c; further analyses in Figure S4b–d).

From these data, we conclude that only minor quantitative differences exist between singlets and doublets concerning the i) host cell binding and ii) TTSS‐1‐dependent entry steps. This hints toward that the preceding flagellar approach might be the main step differentiating doublets from singlets.

### 2.4 Motile doublets swim markedly straighter than singlets

**FIGURE 4:**
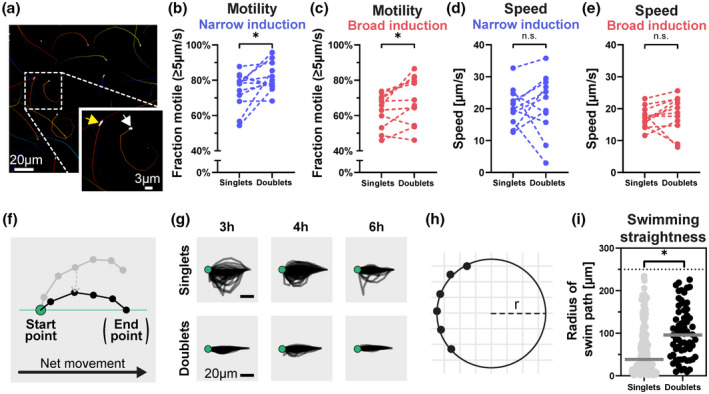
Doublets constitute a straighter‐swimming subpopulation. (a) Representative examples of *S*.Tm^*wt*^ swimming atop glass. Time‐lapse DIC imaging series at 4 h p.sc. (broad induction condition, 1.5 s of imaging), minimally processed to enhance contrast, and tracked using the TrackMate plugin of ImageJ. Insert magnification shows the paths of a tracked singlet (white arrow) and doublet (yellow arrow). (b–e) Comparison of the fraction of motile bacteria (b and c) (defined as a speed >5 μm/s) and the mean speed (d and e) among singlets and doublets. Data from analysis of *S*.Tm^*wt*^ cultured under the narrow (b and d) or broad (c and e) induction condition and analyzed at 3‐6 h p.sc. (Sourced from same experiments as Figure 2b, e), shown as paired comparisons for *n* = 11–12 replicates, pooled from three independent experiments. (f) Conceptual illustration of the transformation applied to track swim paths to align them all at the plot origin and place their end‐point on the x‐axis. Note that intermediate points diverge from the x‐axis dependent on path curvature. (g) Superimposed single‐bacterium swim tracks (6 frames) transformed as in f. Data sourced from three independent experiments at 3‐6 h p.sc. Shown are *n* = 100 randomly chosen tracks per panel. (h) Conceptual illustration for circle‐fitting of the points in a tracked swim path. The radius (*r*) varies with the straightness‐of‐swimming. (i) Quantification of the straightness‐of‐swimming among *S*.Tm^*wt*^ singlets and doublets atop glass, using circle‐fitting as in h. Filled dots show individual measurements, lines represent the medians. Data pooled from three independent experiments (3‐6 h p.sc., broad induction condition; total *n* = 247). Data manually vetted to exclude erroneous fits. Statistical analyses via paired *t* test (b–e) or Mann–Whitney *U* test (i) (*n.s*.: non‐significant; *: *p <* 0.05)

To study single‐bacterium motility dynamics, we revisited the broth culture time‐lapse microscopy datasets (Figure 2b,e), classified the bacteria into singlets or doublets, and quantified their individual swimming behavior (Figure 4a). Under both growth conditions, pair‐wise comparison in the 3‐6 h p.sc. window revealed that doublets were modestly (~1.1‐fold) likelier to be motile than singlets (defined as moving at >5 μm/second; Figure 4b,c). We observed no discernible difference in the average speed under either of the conditions (Figure 4d,e). Therefore, differences in the frequency of motile bacteria and/or average swim speed cannot account for doublet hyperinvasiveness. In further accordance, SEM showed that singlets and doublets featured flagella in a broadly similar distribution along the bacterial body (Figure S5a). However, in the single‐particle tracking analysis, doublet swim paths appeared to follow a noticeably straighter curvature, which we decided to investigate further.

To capture the straightness of individual swim paths, we transformed all paths to start (at t = 0 s) and end (last frame of the path) on the x‐axis of a time plot (Figure 4f). The curvature of the swim path thus corresponds to how much the trajectory diverges from the x‐axis in the intermediate frames of the time‐lapse. When 100 randomly chosen tracks for each category were plotted this way, doublets exhibited markedly straighter swimming (Figure 4g; scored in the relevant 3‐6 h p.sc. window). Since flagellated bacteria generally swim in circular paths near surfaces (Lauga et al., 2006; Park et al., 2019), we next fitted the points of each track to a perfect circle and used the radius of the circle to approximate straightness‐of‐swimming (Figure 4h). Using this approach, singlets were found to swim along a median circle radius of ~39 μm, whereas the median radius for doublets was ~96 μm (Figure 4i; data from 3–6 h p.sc.). Similar conclusions could be drawn from comparing the swim radii of short (bodies ≤2 μm) versus long (bodies >3 μm) bacteria instead of using the waist criterion for categorization (Figure S5b). Hence, the longer *S*.Tm doublets swim on average ~ 2.5‐fold straighter than the shorter singlets. The size of this difference is sufficiently large to account for their hyperinvasive behavior in epithelial cell cultures (Figure 2i,j). However, how straightness‐of‐swimming relates to effective scouting of the epithelial surface remained to be fully assessed.

### 2.5 Doublets survey a higher number of host cells during near‐surface swimming atop enteroid‐derived epithelial monolayers

Singlets and doublets differ predominantly in the curvature of their swimming trajectories (Figure 4). Since breaking out of circular swimming is important for efficient probing of surfaces (Perez Ipiña et al., 2019), we next studied the consequence(s) of the divergent swimming behaviors of the two *S*.Tm categories during epithelial cell layer exploration.

**FIGURE 5:**
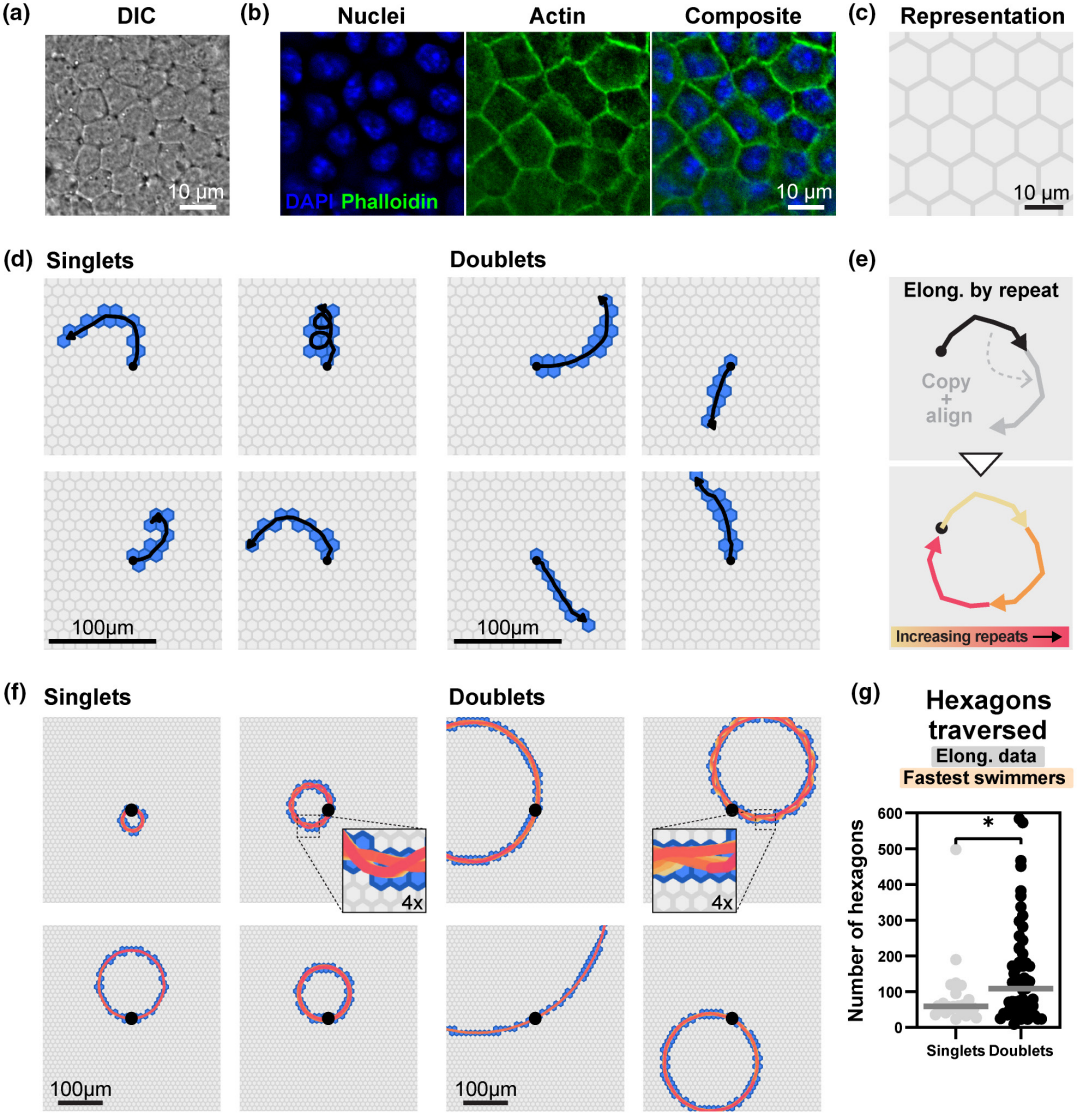
Singlets and doublets exhibit different search patterns atop epithelial cell layers. (a and b) Representative micrographs of a murine enteroid‐derived monolayer, imaged using (a) DIC and (b) fluorescence microscopy. Monolayers were seeded and grown for 3 days, fixed, and stained with DAPI (blue) and Alexa Fluor 488 Phalloidin (green). (c) Stereotypic hexagonal grid representation of the monolayer, used in later analyses. Hexagons have an area corresponding to the experimentally determined mean for individual cells within the monolayer (112.9 μm^2^; *n* = 590 cells). (d) Representative examples of experimentally determined near‐surface swim paths (black arrows) for *S*.Tm^*ΔinvG*^ singlets (left) and doublets (right), superimposed on top of the hexagonal grid representation. Traversed hexagons are highlighted in blue. Data from time‐lapse microscopy (frame interval: 100 ms) of murine enteroid‐derived monolayer co‐incubations. (e) Conceptual illustration for track elongation by repetition, used to create extrapolated swim paths from the time‐restricted experimental data in d. In each iteration, a track (black arrow) is copied (gray arrow), moved to start at the last point of the original track, and rotated so that the direction of the vector formed by its first two points matches the last two points of the original (forming a 0° angle). Tracks were repeated 50 times, forming overlapping paths (thus mitigating differences in track length). (f) Representative examples of extrapolated swim paths for singlets and doublets, generated by elongation of experimentally determined swim paths, as in e. Traversed hexagons are highlighted in blue. (g) Quantification of hexagons traversed by all extrapolated swim paths for *S*.Tm^*ΔinvG*^ singlets and doublets in the group of fastest swimmers (inclusion criteria: speed ≥15 μm/s, tracked for ≥1.5 s; total *n* = 84). Filled dots show individual measurements, lines represent medians. Data pooled from three independent experiments. Statistical analyses via Mann–Whitney *U* test (*: *p* < 0.05). The black dot in panels d–f indicates the path origin. Scale bars in panels a–c: 10 μm, in panels d, f: 100 μm

To assay bacterial motility in this context, we adapted an enteroid‐based intestinal epithelial monolayer model (Samperio Ventayol et al., 2021), cultured in a chamber compatible with time‐lapse DIC imaging from the apical side (van Rijn et al., 2022; see experimental procedures for details). Murine enteroid‐derived epithelial cells cultured in this fashion grew into confluent monolayers with a honeycomb patterning and a high degree of cell packing akin to the intact gut epithelium (Figure 5a,b). We estimated the average cell surface area to 112.9 ± 64.4 μm^2^/cell upon monolayer maturation.

To specifically measure *S*.Tm swimming behavior, these monolayers were incubated with a non‐invasive *S*.Tm strain (lacking InvG, a critical structural component of the TTSS‐1; *S*.Tm^*ΔTTSS−1*^), and observed by DIC microscopy. In this setup, we identified singlets and doublets displaying near‐surface swimming atop the monolayers, with a somewhat increased overall speed in contrast to on glass (Figure S6a; again no significant speed difference between singlets and doublets). Bacteria are commonly trapped in circular/spiraling trajectories, which restricts their exploration capacity (Perez Ipiña et al., 2019), and may limit the number of unique cells they come in contact with. To explore the relationship between straightness‐of‐swimming and the number of traversed host cells, we generated an in silico representation of the monolayer, consisting of a two‐dimensional grid of stereotypical, hexagonal cells with a surface area corresponding to the experimentally measured mean (i.e., 112.9μm^2^/cell; Figure 5c). Individual *S*.Tm near‐surface swim paths, sourced from the monolayer infections, were overlaid on this grid. By highlighting hexagons traversed by each path, a representation of the number of host cells that each one would come into contact with (in the observed time‐span) emerged. This visualized a wide variety of epithelial surface search patterns (Figure 5d). Similar to on glass, doublets were frequently found to traverse the monolayer along straighter trajectories. Fitting singlet and doublet paths to perfect circles, as before, gave an indication that doublets visit a higher number of unique cells during near‐surface‐swimming (Figure S6b).

In contrast to glass, the more complex monolayer surface prompted notable changes in bacterial swimming trajectories due to collisions with debris, shifts in monolayer height, etc. As a consequence, more irregular swim paths were observed atop the monolayer (Figure 5d), which is in line with expectations from earlier literature (Misselwitz et al., 2012; Perez Ipiña et al., 2019). Moreover, *S*.Tm near‐surface swim paths could only be traced for a few seconds atop the monolayers before the bacteria left the focal plane or the field of view. These aspects limited the precision of circular fitting to quantify the outcome of diverse swim patterns. To improve this representation, we instead elongated each path by iteratively copying it 50 times, appending and aligning it to the growing path via vector transformation in each iteration (Figure 5e). This resulted in extrapolated, longer swim paths for each bacterium, which eventually also formed circular patterns (Figure 5f). Quantifying the number of unique traversed cells, as previously, highlighted that extrapolated doublet paths visited on average ~ 1.4‐fold more cells than the corresponding singlet paths (inclusion criteria: speed >5 μm/s; Figure S6c). Notably, when we focused on the fastest swimming *S*.Tm (inclusion criteria: speed >15 μm/s, tracked for >1.5 s, i.e., 15 frames), predicted to represent the first invaders in our invasion assays (Figure 2h–j), this difference grew to ~1.8‐fold in favor of the doublets (Figure 5g).

In summary, from experiments both on glass and atop a real epithelial surface, we conclude that *S*.Tm doublets exhibit a near‐surface swimming behavior biased toward straighter trajectories. This allows them to explore larger swaths of epithelial surface than singlets, which both diversify the total repertoire of swim paths within the S.Tm population and may promote early epithelial cell colonization.

## 3 DISCUSSION

Microbes come in a multitude of shapes and sizes (Chien et al., 2012), and form has proven important in bacterial selection (reviewed in Young, 2006). Recent work demonstrates that the morphology of bacterial pathogens links intimately to their ability to colonize particular host niches. For example, the naturally‐evolved helical shape of *Helicobacter pylori* promotes motility through viscous mucus and early stomach tissue colonization, whereas straight cell‐shaped mutants display an elevated expansion within gastric glands (reviewed in Salama, 2020). A helical or curved shape also impacts motility in viscous media and intestinal colonization by *Campylobacter jejuni* and *Vibrio cholerae* (Bartlett et al., 2017; Stahl et al., 2016). Enterobacteria including *S*.Tm exhibit a straight rod shape as default. However, here we show that heterogeneity in the length of these rods, stemming from natural variation along the bacterial cell cycle, improves this pathogen's capacity to colonize epithelial cells. Hence, cellular morphology can be regarded as a central virulence determinant among pathogenic gut bacteria, which together with the expression of specific virulence factors dictates single‐microbe behaviors during infection.

Rod‐shaped bacteria such as *S*.Tm grow and divide through longitudinal cell elongation followed by the constriction at the middle plane and subsequent fission (Chien et al., 2012). We found that the doublet category, made up of long bacterial bodies anticipating fission, was most abundantly represented during fast exponential growth (i.e., at ~1–3 h p.sc. in rich broth; Figure 2). Here, up to >30% of all bacterial bodies mapped to this category, which is in full agreement with historical observations of enterobacterial morphologies across growth conditions (Kubitschek, 1969). Significantly, however, our analysis revealed a substantial population of *S*.Tm doublets also later, during phases of slowed growth (Figure 2). Comprehensive transcriptional profiling has shown that expression of TTSS‐1‐related genes, i.e., SPI‐1 and non‐SPI‐1‐genes that encode TTSS‐1 effectors, occurs most vigorously in the late exponential phase and early stationary phase (Kröger et al., 2013). This agrees with our single‐cell analysis using a p*sicA*‐GFP reporter (Figure 2). In these phases, *S*.Tm also exhibits flagellar motility and expression of key adhesins, e.g., *SiiE* (Figure 2; Kröger et al., 2013; Main‐Hester et al., 2008; Mouslim & Hughes, 2014), consequently becoming fully equipped for epithelial cell invasion. This intuitively makes sense also when translated to the intact gut. In situations of nutrient excess in the lumen, *S*.Tm appears to prioritize fast planktonic growth, whereas nutrient scarcity and stress promote motility and epithelium‐invasive behavior, which in turn can thwart overall gut homeostasis. Most importantly, our results imply that *S*.Tm population expansion and expression of the epithelial cell‐invasive phenotype should not be regarded as two opposing and chronologically separated states. Rather, the *S*.Tm population shifts gradually from fast growth to virulence and invasiveness, and the overlap between these states generates a transient subpopulation of virulent doublets, shown here to impact epithelial cell colonization.

At the epithelial cell surface, *S*.Tm invasion involves host cell‐binding through flagella, adhesins, and the TTSS‐1 (Collazo & Galán, 1997; Crawford et al., 2010; Gerlach et al., 2007; Horstmann et al., 2020; Lara‐Tejero & Galán, 2009; Li et al., 2019; Misselwitz et al., 2011; Suwandi et al., 2019), and effector‐driven entry (Fattinger et al., 2020; Hardt et al., 1998; Lhocine et al., 2015; Patel & Galán, 2006; Stender et al., 2000; Truong et al., 2013; Zhang et al., 2018). We assessed these steps in isolation. Doublets adhered modestly better to host cells than singlets (~1.2–1.6‐fold better in the absence of flagellar function; Figure 3c; Figure S4b–d), which might be explained by a larger surface area available for interaction. Concerning the entry step itself, doublets elicited similarly sized ruffles as singlets, and also expressed a TTSS‐1 reporter with similar frequency (Figure 3a,b; Figure S4a). Doublets were also commonly found to enter the epithelial cell pole‐first (Figure 1), in effect resulting in that only one half of the body probes its way in (analogous to a singlet), while dragging the other half along. We conclude that the epithelial cell entry‐step occurs through a similar process for singlets and doublets. A doublet entry event will of course result in two sibling bacteria simultaneously colonizing the same or adjacent early SCVs, but the implications of this for the intracellular lifestyle of *S*.Tm, remain to be explored.

Importantly, our combined results point to the preceding step of the flagella‐based approach as the central difference between these morphological *S*.Tm categories (Figure 4 and 5). In our comparisons of flagellar motility patterns, doublets move via a markedly straighter curvature atop surfaces (Figure 4). The most plausible explanation for this phenotype is that the elongated shape of doublets results in a larger average turning radius (Figures S1a,b and S5b). This gains support from independent biophysical modeling studies, which suggest that bacterial swimming radii increase proportionally to cell length and that microbe length affects accumulation at surfaces (Lauga et al., 2006; Shum et al., 2010).

Near‐surface swimming promotes *S*.Tm host cell targeting in tissue culture experiments (Misselwitz et al., 2012; Vonaesch et al., 2013), and also occurs atop the gut mucus layer in vivo (Furter et al., 2019). In co‐cultures with epithelial cells, this exploratory *S*.Tm behavior has recently been proposed to proceed by a random search, independent from chemotaxis (Otte et al., 2021). That study further highlights a remarkable spread in search patterns among single *S*.Tm particles (Otte et al., 2021). It appears plausible that the total surface exploration capacity of the pathogen population benefits from a high degree of variability between individual bacteria. The presence of *S*.Tm of different lengths (i.e., singlets, doublets, and intermediates between these forms) within the surface‐exploring population could be a key source of such variability. Another source may be the phase shifts between expression of either FljB or FliC as the flagellar filament subunit, which occur over longer time scales and have been shown to affect *S*.Tm motility patterns (Horstmann et al., 2017; Yamaguchi et al., 2020). Taken together, large heterogeneity in near‐surface search behaviors likely aids in maximizing the chances that a sub‐fraction of the *S*.Tm population finds cracks in the mucus layer (Furter et al., 2019), and arrives at favorable sites for epithelial cell invasion. Our data suggest that the *S*.Tm doublet category may be particularly good at exploring larger swathes of epithelial surface in this process (Figures 4 and 5).

Bacterial pathogen behaviors have historically been studied by bulk assays. More recently, single‐cell approaches have revealed examples of remarkable microbial heterogeneity during host cell interaction. For example, sensing of epithelial surfaces by *Pseudomonas aeruginosa* generates two subpopulations which colonize the local surface or explore more distant regions, respectively (Armbruster et al., 2019; Laventie et al., 2019). Cell‐to‐cell spread of intracellular *Listeria monocytogenes* within an epithelial cell layer similarly relies on a small subpopulation of pioneering bacteria (Ortega et al., 2019). Intrapopulation heterogeneity also includes bistable expression or phase variation of many of the virulence factors for epithelial cell invasion, including the TTSS‐1, flagella, and main adhesins of *S*.Tm (García‐Pastor et al., 2019). Our present work adds bacterial cell length heterogeneity to this list of parameters that impact key infection cycle step(s), and emphasizes the need for a close‐up view to fully understand bacterial pathogenesis.

## 4 EXPERIMENTAL PROCEDURES

### 4.1 *Salmonella* Typhimurium strains, plasmids, and culture conditions

All strains used in this study were derivatives of *Salmonella enterica* serovar Typhimurium (*S*.Tm) SL1344 (*S*.Tm^*wt*^; SB300; streptomycin‐resistant; Hoiseth & Stocker, 1981). Derivatives were *S*.Tm^*ΔTTSS−1*^ (Δ*invG*; Kaniga et al., 1994), *S*.Tm^*ΔmotA*^ (Geiser et al., 2021), and *S*.Tm^*ΔfliCΔfljB*^ (Samperio Ventayol et al., 2021). Where indicated, strains transformed with plasmids p*rpsM*‐GFPmut2 (pM965; Stecher et al., 2004), p*rpsM*‐mCherry (pFPV‐mCherry, Addgene plasmid #20956; Drecktrah et al., 2008), or p*sicA*‐GFP (pM972; Sturm et al., 2011) were used. Bacterial cultures were grown under either “narrow” or “broad” induction conditions, to elicit either growth phase‐specific or ‐independent virulence gene expression, respectively. The “narrow” condition cultures were grown for 24 h in Luria Broth (LB, 0.1 M NaCl; Sigma‐Aldrich) with appropriate antibiotics, followed by sub‐culturing at a 1:100 dilution, for 0–24 h (depending on the experiment). In contrast, the “broad” condition cultures were grown in LB/0.3 M NaCl for 12 h, followed by sub‐culturing at a 1:20 dilution, for 0–24 h in the same medium (depending on the experiment). When required, streptomycin (final concentration: 50 μg/ml; Sigma–Aldrich) or ampicillin (50 μg/ml; Sigma–Aldrich) was added to ON cultures. Sub‐cultures were antibiotics‐free. All cultures were grown at 37°C in a roller‐drum incubator to ensure oxygenation.

### 4.2 Epithelial cell line culture maintenance

The HeLa (human epithelial cells; CCL‐2, ATCC) and m‐IC_cl2_ cell lines (murine small intestinal epithelial cells; Bens et al., 1996) were cultured as previously described (Di Martino et al., 2019; Fattinger et al., 2020). Both cell lines were passaged 2–3 times per week. Briefly, HeLa cells were maintained at 37°C and 10% CO_2_ in high‐glucose Dulbecco's modified Eagle's medium (DMEM; Gibco), supplemented with 10% heat‐inactivated fetal bovine serum (FBS; Thermo Fisher Scientific) and 100 U/ml PenStrep (Gibco). m‐IC_cl2_ cells were maintained at 37°C and 5% CO_2_ in DMEM/F12 (Invitrogen) supplemented with 2% heat‐inactivated FBS (Invitrogen), 100 U/ml PenStrep, 5 μg/ml insulin (Invitrogen), 50 nM dexamethasone (Sigma–Aldrich), 60 nM sodium selenite (Sigma–Aldrich), 5 μg/ml bovine apo‐transferrin (Sigma–Aldrich), 1 nM triiodothyronine (Sigma–Aldrich), 60 ng/ml EGF (Sigma–Aldrich), 2 mM glutamine (Invitrogen), 12.5 mM D‐glucose (Sigma–Aldrich), and 20 mM HEPES (Gibco). For infection experiments, antibiotics were omitted and cells were seeded onto the relevant cell plastics 24 h before infection.

### 4.3 Light microscopy

Several microscope setups were used in the study. Predominantly, we used a custom‐built microscope, based on a Nikon Eclipse Ti2 body equipped with a 60x Plan Apo Lambda air objective (0.7 numerical aperture, NA; 183 nm final pixel size; Nikon) and a 100x Plan Apo oil objective (1.45 NA; 110 nm final pixel size; Nikon), and a back‐lit sCMOS camera (Prime 95B, Photometrics). Bright‐field images were collected using differential interference contrast (DIC), while fluorescence was excited using a Spectra‐X light engine (Lumencor). This microscope was used for imaging of all bacterial cultures, live HeLa cell infections, and quantitative imaging of fixed HeLa samples. Alternatively, we used a second custom‐built microscope with a dipping‐objective configuration, based on a heated 60x CFI APO NIR water‐dipping objective (1.0 NA, 2.8 mm working distance; final pixel size of 108 nm; Nikon) and a D‐CUO DIC Oil Condenser (1.4 NA; Nikon) on a Thorlabs Cerna upright microscopy system. Images from this system were acquired with an ORCA‐Fusion camera (Hamamatsu photonics). This microscope was used for live enteroid‐derived monolayer imaging. Both microscopes were controlled by μManager 2.0‐gamma (Edelstein et al., 2014), and live samples were maintained on the stage at 37°C in a moisturized CO_2_‐controlled atmosphere. For imaging of mouse intestinal tissue sections, we used a Zeiss Axiovert 200 m microscope with 10x‐100x objectives, a spinning disc module (Visitron), and two Evolve 512 EMCCD cameras (Photometrics). Additional qualitative imaging of fixed HeLa cells was done using a Zeiss LSM700 point‐scanning confocal system (BioVis imaging facility, SciLifeLab, Sweden). All images were analyzed using ImageJ (Fiji distribution), Visiview (Visitron), and/or the OMERO server interface. For high‐frequency microscopy image stacks, frame interval correction was applied as described previously (Eriksson et al., 2021).

### 4.4 Field emission scanning electron microscopy

SEM of *S*.Tm inocula, HeLa cells, m‐IC_cl2_ cells, and mouse intestine samples was performed as described previously (Fattinger et al., 2020). Briefly, samples were fixed in 2.5% glutaraldehyde (Polyscience), washed in Krebs‐Ringer buffer or PBS, and treated with 1% OsO_4_ (Polyscience). HeLa and m‐IC_cl2_ cells were additionally incubated in 0.5% carbohydrazide and treated with 1% OsO_4_ for a second time. All samples were incubated in acetone before dehydration and critical‐point‐drying by liquid CO_2_ using an Autosamdri‐931 (Tousimis or Bal‐Tec CPD030). Samples were mounted on aluminum SEM stubs, sputter‐coated with 5 nm platinum/palladium (Safematic CCU‐010 or Bal‐Tec SCD500). Samples were explored using a Zeiss Merlin Gemini II ultra‐high resolution field emission scanning electron microscope (acceleration voltage 5 kV) and images were captured and analyzed with Zeiss SmartSEM and ImageJ. Where relevant, pseudo‐coloring was applied post‐processing in Adobe Illustrator.

### 4.5 Imaging of live bacteria

For live microscopy of *S*.Tm, cultures at the indicated time point p.sc. were diluted as indicated and added to black‐walled glass‐bottomed 96‐well plates (glass thickness #1.5H, CellVis). When relevant, an excess of carbonyl cyanide 3‐chlorophenylhydrazone (CCCP; Sigma C2759) was added to instantaneously inhibit flagella‐based motility (final concentration: 10 mM). To limit the depth‐axis movement of bacteria during imaging, a low‐volume solution (40‐50 μl) was used and carefully stretched out to form a thin film covering the bottom. DIC and/or fluorescence imaging was done at 60x magnification, immediately upon addition to the plate (and/or after further incubation, as required). For SPI‐1 expression quantification of the *S*.Tm/p*sicA*‐GFP reporter strain, fluorescence excited at 475 nm was collected for 100 ms. For general inoculum imaging, the sample was exposed using DIC for 10‐40 ms at 250 ms intervals for 12 frames (= 3 s). Imaging was completed within 5 min at each time point to limit the long‐term effects of dilutions and exposure to CCCP. Classification of *S*.Tm into singlet and doublet categories by all imaging modalities was based on the absence/presence of a visible waist. The categorization was done manually, with key data sets validated by an additional blinded observer and/or reassessment based solely on bacterial body length measurements (e.g., Figure S5b). Automated assessment of intensity profiles across DIC images of singlets and doublets was used as a final way of vetting the rigor of the classification (Figure S1a,b).

### 4.6 Imaging of HeLa cell infections

For live infection imaging, HeLa cells were seeded at a ~ 80% confluency in multi‐well glass‐bottom plates (#1.5H; Cellvis). Inocula were created by diluting the sub‐cultures to an approximate MOI = 20 in the condition‐specific medium. A fraction of the inoculum was further diluted to 50–200 bacteria per field of view and imaged in empty wells, similarly to for other bacterial cultures (see above). A second fraction of the inoculum was added to the HeLa cells and DIC and/or fluorescence imaging was started immediately (<10 min after set time). Cells for fixation were grown in either multi‐well glass‐bottomed plates or on glass microscopy slides with detachable 8‐well walls (#1.5H; MatTek) for short‐ or long‐term storage, respectively. Samples were washed three times with warm medium and fixed in 2% paraformaldehyde (PFA; Sigma–Aldrich) in phosphate‐buffered saline (PBS; Gibco) for 15 min, washed with PBS, and permeabilized using 0.1% Triton‐X (Sigma–Aldrich) for 15 min. Samples were washed once more, blocked using PBS/2% bovine serum albumin (BSA, Fischer Scientific), and incubated with anti‐LPS primary antibodies (rabbit *Salmonella* O Antiserum Factor 5; Difco) and Cy3‐conjugated secondary antibodies (Goat‐α‐rabbit‐IgG; Fischer scientific) for 1 h each. Lastly, samples were stained with DAPI (1:1000; Sigma–Aldrich) and Alexa Fluor 488 Phalloidin (1:25–1:50; Molecular Probes) in PBS for 30 min, washed, and analyzed. For long‐term storage, well walls were detached and samples mounted in Mowiol (Calbiochem).

### 4.7 Adhesion assay

In preparation for adhesion assays, HeLa cells were seeded at ≥95% confluence ON in a glass‐bottomed 6‐well plate (#1.5H; CellVis). The bacterial inoculum of the indicated strain was diluted to MOI = 60 in a HeLa cell medium lacking antibiotics and added to the wells. If indicated (“spin” in figures), the plate was first centrifuged at 700 × g for 5 min at room temperature. All samples were then incubated at 37°C and 10% CO_2_ for a total infection time of 10 min (including centrifugation). During this time, the inoculum was imaged separately, as described above. After the co‐incubation, the cell layer was washed once with a pre‐warmed medium and fixed in‐plate for 15 min in 2% PFA. The plate was washed once more, kept in PBS, and imaged using DIC and mCherry emission at 575 nm, in multiple fields‐of‐view per sample.

### 4.8 Murine enteroid‐derived epithelial monolayers

Murine small intestinal enteroid cultures were thawed from previously established stocks (Hausmann et al., 2020), and maintained within Matrigel (Corning) domes overlaid with complete mouse IntestiCult (Stemcell), as detailed elsewhere (Samperio Ventayol et al., 2021). Enteroid‐derived monolayers were established using an adapted version of a recently developed 3D‐printed polylactide (PLA) transwell chamber, allowing a short working distance for dipping‐objective microscopy (van Rijn et al., 2022). Here, instead of an alumina‐based bottom membrane, the chamber was equipped with a high‐attachment polyester membrane (Thermanox™; Thermo Fischer). Transwells were assembled, and membranes were coated ON with 75 μg/ml Poly‐L‐Lysine (Sigma–Aldrich), washed three times with PBS, and air‐dried. A neutralized collagen‐1 (Corning) hydrogel solution was prepared as described (Samperio Ventayol et al., 2021) and pipetted onto the poly‐L‐lysine‐coated membranes, which were then incubated at 37°C for 1.5 h. Enteroid cultures, pre‐treated in complete mouse IntestiCult supplemented with 3 μM CHIR99021 (Cayman Chemical) and 1 mM valproic acid (Cayman Chemical), were disrupted into a single‐cell suspension by incubation in Gentle Cell Dissociation Reagent (Stemcell) and passing the solution through a G25 needle ≥10 times. Cells were counted in a Bürker chamber and ~ 60.000 cells/well seeded onto the collagen‐1 hydrogel. The cultures were maintained in IntestiCult/CHIR99021/VPA further supplemented with 10 μM Y‐27632 (Sigma–Aldrich) for 24 h, and thereafter in IntestiCult until day three by which they achieved ≥95% confluence and formed a honeycomb pattern, and were thereafter used for bacterial motility experiments as detailed below.

### 4.9 Analysis of *S*.Tm swim path trajectories atop glass

Raw microscopy movies from live bacterial cultures on glass were first cut to only include a short timeframe (first 1.5 s), to limit bias toward incoming bacteria appearing after the start of imaging. Then, movies were imported into ImageJ and automatically corrected for uneven illumination, temporal mean subtraction, and background subtraction. Subsequently, tracking of single‐bacterium swim paths were carried out using the TrackMate plugin (Tinevez et al., 2017) of ImageJ, before downstream analysis in R. Briefly, bacteria were assigned as either singlets and doublets, and swim speed, fraction motile bacteria (i.e., mean speed ≥5 μm/s), and doublet frequency was quantified. Other analyses included both qualitative and quantitative assessments of straightness‐of‐swimming. A qualitative assessment was implemented by transforming the first point of all tracks to Origo and then rotating tracks so that their last point landed on the x‐axis. This made it possible to visually compare swim path curvatures between categories. Quantification of straightness‐of‐swimming was done by comparing the radius of a circle fitted to the points of each swim path individually. This analysis employed iterative algebraic optimization via the *circlefit* function of the *pracma* R package.

### 4.10 Assessing the number of cells visited by *S*.Tm atop murine enteroid‐derived monolayers

Enteroid‐derived monolayers were infected with *S*.Tm^*ΔinvG*^ (broad induction condition). A single image of the monolayer and a movie of swimming bacteria directly above it was captured using DIC with an exposure time of 10 ms. Movies of bacteria were captured at 250 ms intervals for in total of 300 frames (30s). Superimposing each movie on the underlying monolayer could visualize the concept, but not allow stringent quantification of the number of cells traversed by each bacterium during short near‐surface swimming bouts. Instead, a stereotypic hexagonal grid was constructed in silico, using hexagons of the same area as the average cell in the monolayer images (A = 112.9 μm^2^). Straightness‐of‐swimming (of tracks from analysis in TrackMate) was analyzed as described for tracks on the glass above. Actual tracks, or circular fits of mean tracks (as indicated in figure legends), were superimposed on the hexagonal grids. Extrapolation of tracks by elongation was done via an iterative process, repeating the same track 50 times head‐to‐tail, and rotating/transforming each iteration of the track to align with the previous, using the *matlib* R package (Hunter, 2007). This alignment was defined so that: (a) the first point of the current iteration was located on the same coordinates as the last point of the previous, and (b) the vector of the first two points in the current iteration (points 1➔2) aligned with the vector of the last two points of the previous (points (n‐1)➔(n); i.e., maintaining the rotational angle, so that the track initially “continued” in the same direction). The elongated paths were superimposed on the hexagonal grid pattern, and the number of traversed hexagons was automatically enumerated and compared between singlets and doublets (inclusion criterion: swim speed ≥5 μm/s). “First invaders” (high‐speed swimmers) were also compared separately (inclusion criteria: tracked for ≥1.5 s, swim speed ≥15 μm/s).

### 4.11 Mice and in vivo infections

Mice were kept in individually ventilated cages in specific pathogen‐free settings (RCHCI and EPIC facilities, ETH Zürich). Wild‐type C57BL/6 mice were originally from Charles River, *Nlrc4* ^*−/−*^ mice from (Mariathasan et al., 2004). Infections were performed as detailed previously (Barthel et al., 2003). In short, 8–15‐week‐old mice were treated with 25 mg streptomycin sulfate (Applichem) per oral gavage. 24 h later, mice were infected per oral gavage with 5 × 10^7^ CFUs of *S*.Tm. At 8‐9 h p.i. the cecae were excised and opened up, the mucosal tissue was washed extensively to remove luminal bacteria, and the tissue was fixed in 4% PFA, saturated in 20% sucrose, and frozen in optimum cutting temperature medium (OCT; Tissue‐Tek) before cryo‐sectioning. 10 μm cross‐sections were air‐dried and stained with anti‐LPS primary antibodies and Cy3‐conjugated secondary antibodies (Bethyl Laboratories) for ~1 h each, followed by counter‐staining using DAPI (Sigma–Aldrich) and Alexa Fluor 488 Phalloidin (Molecular probes) and mounted in Mowiol. Scoring of *S*.Tm subpopulations was done manually, differentiating singlets/doublets by length and/or the presence of a visible waist, similar to for the inocula and HeLa cell culture infections.

### 4.12 Statistical analysis

Data management, plotting, and statistical analysis were handled in R (v4.0.4; www.r‐project.org), using R studio (v1.4.1106; www.rstudio.com). The main packages of functions used in R were *tidyverse* (v1.3)*, pracma* (v2.3.3), *matlib* (v0.9.4), and *ggplot2* (v3.3.3), supplemented by functions from the *reshape2, purrr, boot, patchwork, knitr, ggforce, ggpubr, ggbeeswarm, gridExtra, and colorspace* libraries. Further management, plotting, and analyses were done using GraphPad Prism (version 8–9; www.graphpad.com). Where appropriate, statistical significance was evaluated by Mann–Whitney *U* test or paired *t* test, both with base alpha = 0.05 (* p < 0.05; ** p < 0.01).

## CONFLICT OF INTEREST

The authors declare no competing interests.

## AUTHOR CONTRIBUTIONS

Viktor Ek and Mikael E. Sellin conceived the study; Viktor Ek, Stefan A. Fattinger, Alexandra Florbrant, Wolf‐Dietrich Hardt, Maria Letizia Di Martino, Jens Eriksson, and Mikael E. Sellin designed research; Viktor Ek, Stefan A. Fattinger, and Alexandra Florbrant performed research and analyses; Jens Eriksson and Maria Letizia Di Martino provided research tools and reagents. Mikael E. Sellin acquired funding. Viktor Ek and Mikael E. Sellin wrote the paper. All authors reviewed and edited the paper.

## ETHICS APPROVAL

Animal experimentation was performed in accordance with the Swiss Federal Government guidelines of the animal experimentation law (SR 455.163 TVV). The protocols used were approved by the Cantonal Veterinary Office of the canton Zürich, Switzerland (Kantonales Veterinäramt ZH license 193/2016 and 158/2019).

## Supporting information


Figure S1‐S6

Video S1

Video S2

## Data Availability

All data are presented within the manuscript and the Supporting Information.
