# Supplementary material for: A motile doublet form of Salmonella Typhimurium diversifies target search behavior at the epithelial surface
Source: Mol Microbiol. 2022 Apr 12;117(5):1156–72. doi: 10.1111/mmi.14898 (PMC9325389; doi:10.1111/mmi.14898)
Supplement: Supplementary file 1 — Figure S1‐S6 [file MMI-117-1156-s003.pdf]

Supplementary information:
A Motile Doublet Form of *Salmonella* Typhimurium Diversifies Target Search Behaviour at the Epithelial Surface

Viktor Ek[1], Stefan A. Fattinger[1,2], Alexandra Florbrant[1], Wolf-Dietrich Hardt[2], Maria Letizia Di Martino[1], Jens Eriksson[1], Mikael E. Sellin[1#]

AFFILIATIONS

1 Science for Life Laboratory, Department of Medical Biochemistry and Microbiology, Uppsala University, Sweden.

2 Institute of Microbiology, Department of Biology, ETH Zurich, Zurich, Switzerland

[#]Correspondence: mikael.sellin@imbim.uu.se

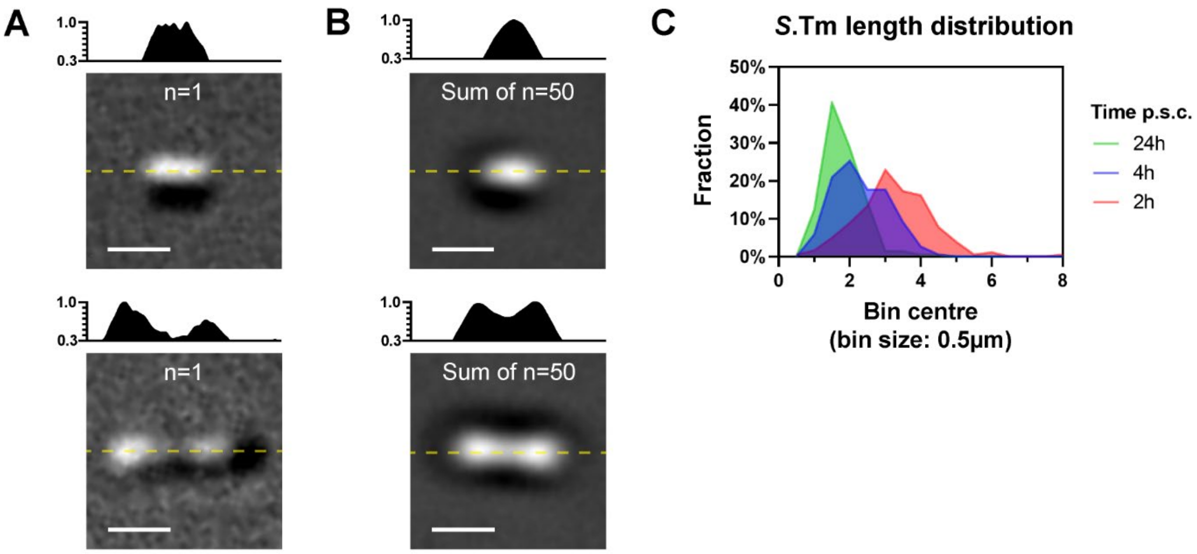

Figure S1. DIC intensity profiles and length distribution of *S.*Tm^{wt} populations

(A-B) Representative DIC intensity profiles of sample micrographs for individual singlets and doublets (A, *n*=1), as well as a representative projection for each subpopulation, representing the sum of *n*=50 randomly picked images in each case (B). The manual classification of bacteria into singlets/doublets was based on the presence of a visible "waist" as exemplified here. Cultures were grown under the broad induction condition before imaging at 4h p.sc. (C) *S.*Tm^{wt} were grown under the narrow induction condition and the lengths of bacterial particles at 2h (n=179), 4h (n=186), or 24h (n=209) p.sc. were quantified by DIC microscopy and manual measurements in Image J. Data pooled from three independent experiments, and binned with a bin size of 0.5µm centred around each tick on the x-axis (0.25 on each side; values in the range 3.75-4.24 are binned to 4 et.c.). Scale bars: 2µm.

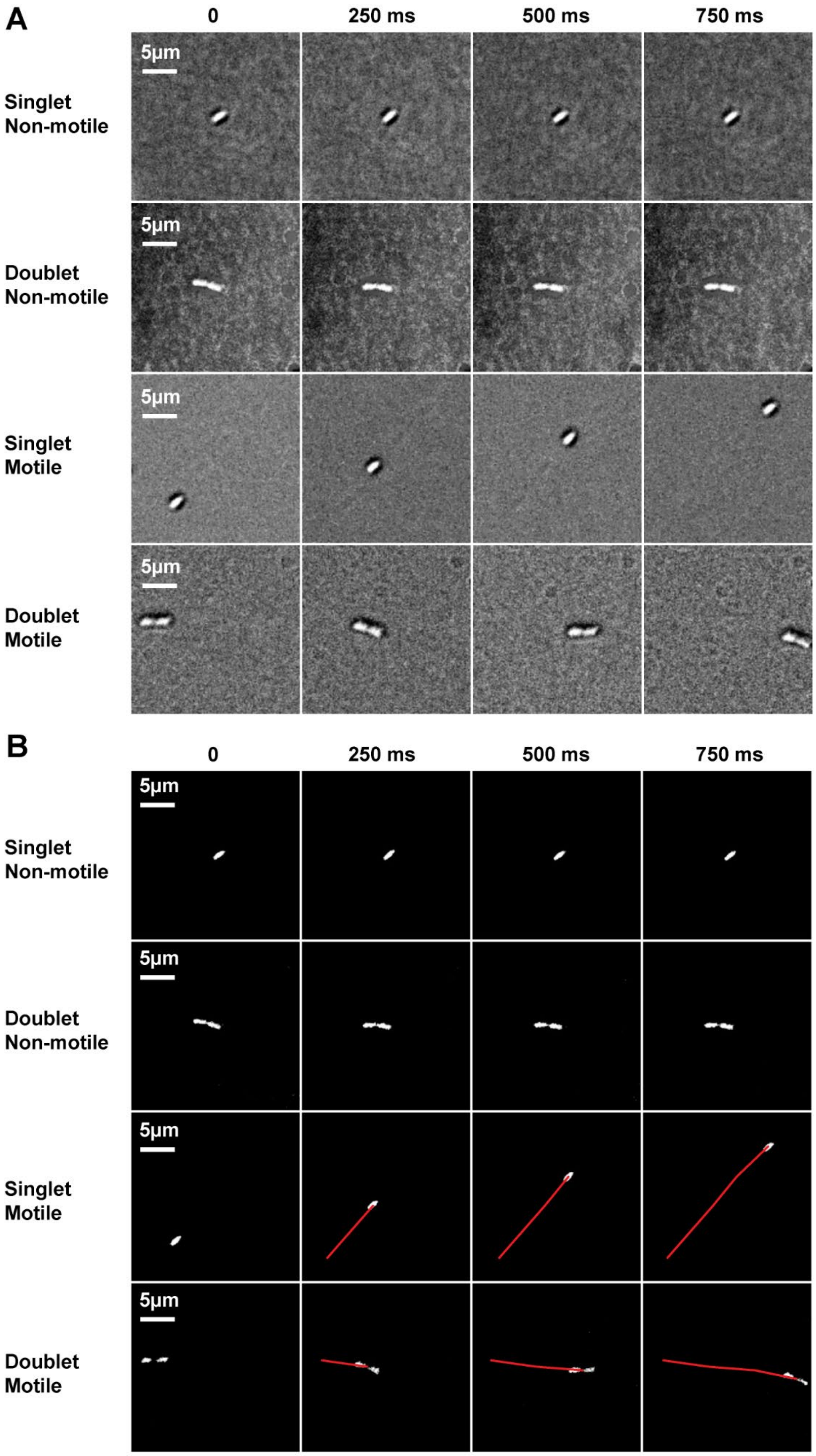

A
0
250 ms
500 ms
750 ms
5µm
Singlet
Non-motile
Doublet
Non-motile
Singlet
Motile
Doublet
Motile
B

Figure S2. Analysis of *S.*Tm singlet and doublet motility by DIC time-lapse microscopy and single-particle tracking

*S.*Tm^{wt} were grown under the broad induction condition and imaged by time-lapse DIC microscopy at 4h p.sc. (A) Representative time-lapse images of a non-motile (speed <5 µm/s) and motile singlet and doublet. Images processed for uneven illumination correction and light contrast enhancement. (B) Corresponding images after complete processing (see experimental procedures for details) and including tracked swim paths (in red, for motile bacteria). Time intervals (in milliseconds; ms) indicated atop panels. Scale bars: 5µm.

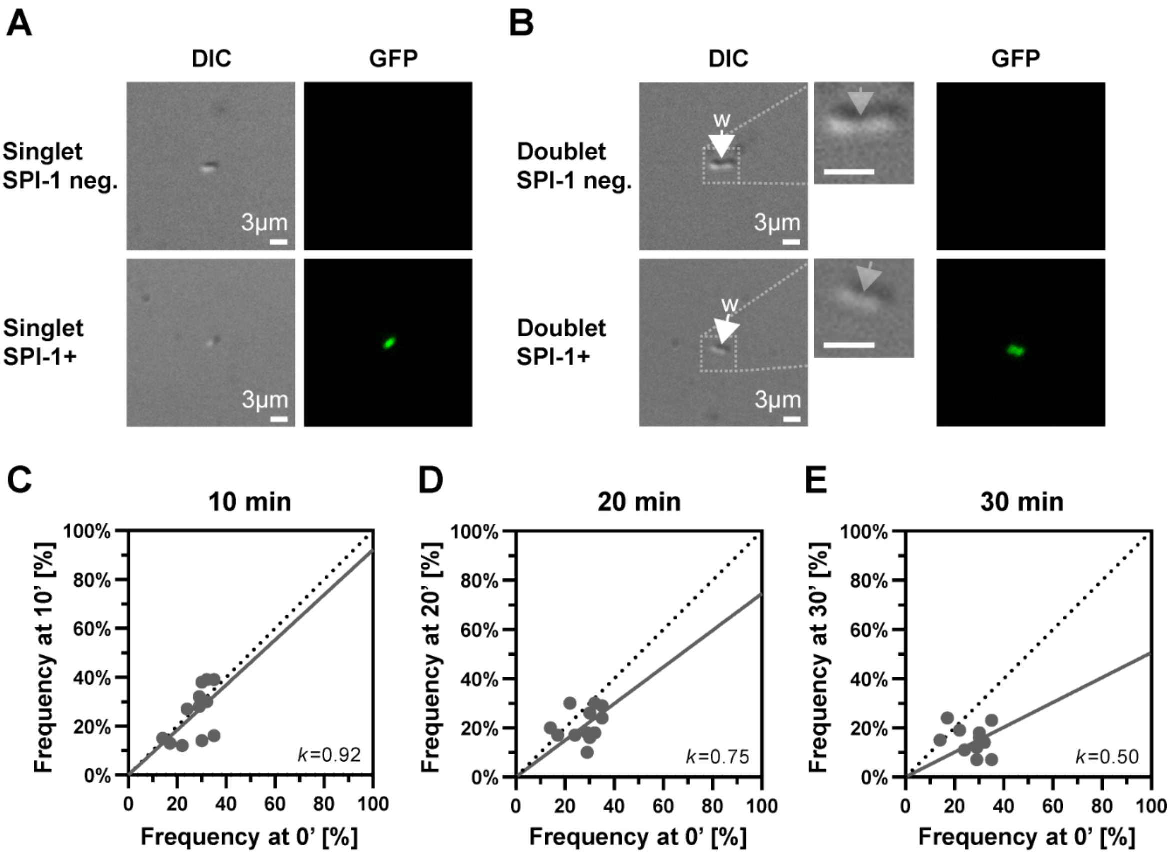

Figure S3. Scoring of SPI-1/TTSS-1 expression and doublet frequency dynamics in tissue culture medium

(A-B) Scoring of TTSS-1-expressing singlets and doublets with the *S.*Tm/p*sicA*-GFP reporter strain. *S.*Tm/p*sicA*-GFP were grown under the broad induction condition and imaged by DIC and fluorescence microscopy at 4h p.sc. Shown are representative DIC and fluorescence (GFP; green) image examples for singlets (A) and doublets (B). DIC images of the doublet are also shown enlarged (magnification ~3x). "W" and/or arrow denotes the waist of doublets. Scale bars: 3µm. (C-E) Doublet frequency within the *S.*Tm population upon transfer of the inoculum into tissue culture medium. *S.*Tm^{wt} were grown under the broad induction condition, and the inoculum at 4h p.sc. was incubated in tissue culture medium (high-glucose DMEM/10% FBS) in the heated microscope enclosure. Doublet frequency was quantified from DIC images immediately in the inoculum, and after (C) 10min, (D) 20min, or (E) 30min (*i.e.* the longest infection time used in other assays) of adding the inoculum into the tissue culture medium.

Graphs show frequency of doublets in the inoculum (x-axis; 0 min) vs. the respective time-point (y-axis). Black dotted line illustrates a theoretical 1:1 ratio (k=1). Data points shown as filled circles with a linear regression line (grey; k-values indicated). Note that exposure to the tissue culture medium does not lead to an increased frequency of doublets, but rather a modest decrease, compared to in the inoculum (0 min). Data pooled from three experiments (n=12).

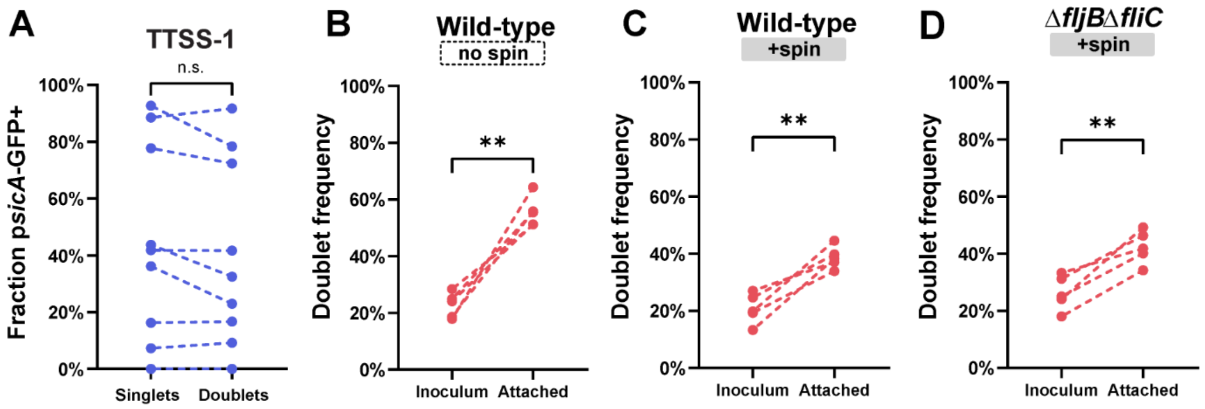

Figure S4. TTSS-1 expression in singlets and doublets during growth under the narrow induction condition and further assessment of host cell adhesion

(A) Comparison between *S*.Tm singlet and doublet subpopulations in the expression of TTSS-1, using the p*sicA*-GFP reporter strain cultured under the narrow induction condition and analysed at 3-6h p.sc. (sourced from same experiments as Fig. 2C). Data shown as paired comparisons for n=9 replicates, pooled from three independent experiments. (B-D) Additional comparisons of doublet frequencies in inocula and in the populations attaching to HeLa cells. HeLa cells were treated with Cytochalasin D and co-incubated for 10min with pFPV-mCherry-carrying inocula (broad induction condition, 4h p.sc.) of either *S*.Tm^{wt} (B-C) or a non-flagellated strain (*S*.Tm$^{\Delta fliC\Delta fljB}$; D), with or without a spin step to promote contact as indicated atop graphs. Cells were washed and the attached bacteria categorized. Data shown as paired comparisons for n=5 independent experiments in each case. Statistical analyses via paired t test (*n.s.*: non-significant; **: p<0.01). Interpretation: The results in B-D show that under conditions where *S*.Tm^{wt} rely strictly on flagellar motility to reach the target cells (i.e. B), doublets are markedly enriched on the host cell surface (~2.4-fold). Introducing a spin step diminishes the need for flagellar motility, which makes this difference less pronounced (~1.9-fold; C). Finally, a strain completely lacking flagella, which are also important for adhesion, reveals that doublets still adhere moderately better (~1.6-fold) than singlets to the host cell surface (D). Hence, doublets are superior to singlets both with respect to flagellar approach and host cell surface binding.

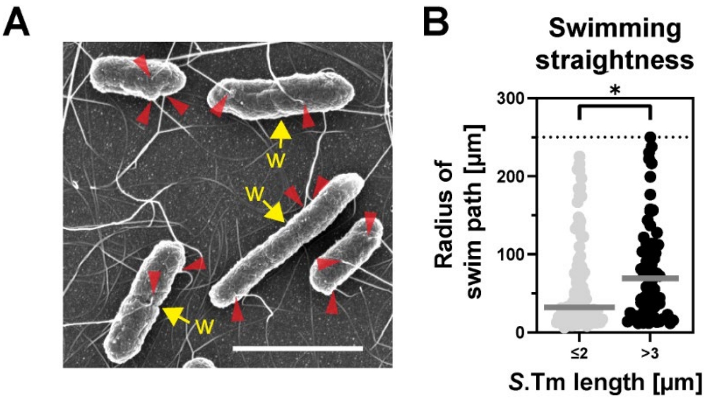

Figure S5. Flagellar appearance on singlets/doublets and the swim radius dependence on body length

(A) Representative SEM micrograph of *S.*Tm^{wt} showing similar flagellar appearance and distribution between singlets and doublets. "W" and arrow denotes the waists of doublets, and red arrowheads denote bases of bacterium-connected flagella. Scale bar: 2µm. (B) Quantification of the straightness-of-swimming among *S.*Tm^{wt} bacteria atop glass, using circle-fitting (as previously; see Fig. 4). The graph shows a comparison between categories of bacteria with a length of ≤2µm versus >3 µm. Filled dots show individual measurements, lines represent the medians. Data pooled from three independent experiments (3-6h p.sc., broad induction condition; total n=163). Statistical analysis via Mann-Whitney U test (*: p<0.05).

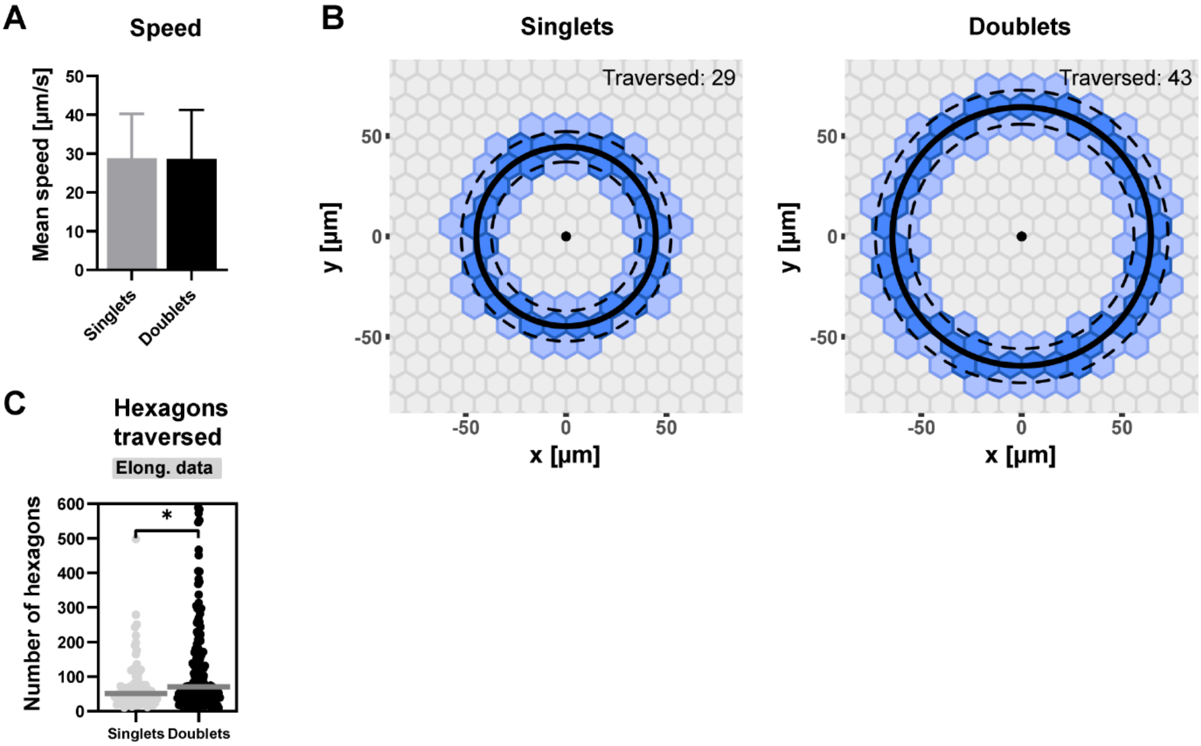

Figure S6. Further comparisons of the divergent search patterns of singlets and doublets atop epithelial cell layers

*S.*Tm$^{\Delta invG}$ were grown under the broad induction condition, and the inoculum at 4h p.sc. was added to murine enteroid-derived monolayers and observed using DIC microscopy (frame

interval: 100ms). After contrast enhancement, near-surface swim paths of individual bacteria were tracked and analysed. Data from three independent experiments (*n*=250 total bacteria, based on data in Fig. 5D, G; inclusion criterion: speed >5µm/s). (A) Quantification of mean speed among singlets and doublets in the population on the monolayer. (B) Comparison of circles fitted to swim paths for singlet and doublet bacteria atop the monolayers. Circles radii represent the mean radii of circles fitted to each swim path, and hexagons traversed by this path are coloured dark blue (singlets: 29 hexagons; doublets: 43 hexagons). Dashed circles represent 95% CI (light blue hexagons). (C) Hexagons traversed by elongated tracks among singlets and doublets. After tracking, iterative elongation of tracks was done for 50 iterations. Filled dots show individual measurements, lines represent medians. Statistical analysis via Mann-Whitney *U* test (*: $p<0.05$).

Video S1. Analysis of *S.*Tm motility by time-lapse DIC imaging and single-particle tracking

Example movie from bacterial tracking experiments. *S.*Tm^{wt} were grown under the broad induction condition and imaged atop glass by time-lapse DIC microscopy at 4h p.s.c (250ms intervals; 3s duration). Time-lapse stacks were processed using ImageJ, and single particles tracked via TrackMate. Scale bar: 9µm.

Video S2. *S.*Tm doublet invasion of an epithelial cell

Example movie of an *S.*Tm doublet invading a HeLa cell through ruffle formation, and subsequently dividing. *S.*Tm/p*rpsM*-GFP were grown under the broad induction condition and added to a culture of HeLa cells at 4h p.sc. The infection was imaged by time-lapse DIC and fluorescence (GFP; green) microscopy (30s intervals; 15min duration), and time-lapse stacks processed using ImageJ. Shown is a magnified view of one invasion event. Scale bar: 3µm.
